# Is Adolescent Risk Behavior Associated With Cross-Household Family Complexity? An Analysis of Post-separation Families in 42 Countries

**DOI:** 10.3389/fsoc.2022.802590

**Published:** 2022-02-16

**Authors:** Sebastian Schnettler, Anja Steinbach

**Affiliations:** ^1^Department of Sociology, University of Oldenburg, Oldenburg, Germany; ^2^Department of Sociology, University of Duisburg-Essen, Duisburg, Germany

**Keywords:** adolescence, divorce, health, HBSC, risk behavior, separation, stepfamilies, family complexity

## Abstract

We examine whether complex cross-household structures of post-separation families are associated with higher risk-taking behavior in adolescence (substance use, bullying, early sexual onset) and whether the proportion, and thus statistical normality, of complex family types in a certain country is a potential moderator of this association. Drawing on representative data from 42 countries and regions from the Health Behavior in School-aged Children (HBSC) study in 2001, 2006, and 2010 (*N* = 506,977), we provide detailed analyses on adolescent risk behavior even for very rare family types, thereby accounting for the complex cross-household structure present in many post-separation families. We combine logistic and count regression models to analyze risk incidence and intensity. Controlling for relevant child and family characteristics, our results reveal a gradient along which adolescent risk-taking increases with family complexity: The incidence and intensity of risk-taking among adolescents is lowest in two-biological-parent and highest in two-household families with stepparents in both households. The association decreases with a higher proportion of the respective family type in a country. However, the differences between family types, other than the two-biological parent family, are not as pronounced as expected.

## Introduction

Risk behavior among adolescents is associated with reduced mental wellbeing, physical health, and academic achievement that can persist well into adulthood (e.g., Hurrelmann and Richter, 2006). One factor that has been repeatedly found to be significantly associated with risk behavior is family disruption and related family transitions (McArdle et al., 2002; Bjarnason et al., 2003; Griesbach et al., 2003; Barrett and Turner, 2006; Brown and Rinelli, 2010; Fomby and Sennott, 2013; Rüütel et al., 2014). Single family transitions like divorce and re-partnering, as well as some of their consequences have been studied extensively (Thomson, 2014; Hadfield et al., 2018). Previous research reveals an increased prevalence of risk behavior in single parent and stepfamilies in comparison to adolescents who are growing up in two-parent-biological families, even after controlling for several other relevant factors (Griesbach et al., 2003) and when studied longitudinally, comparing the same individuals as they transition into a stepfamily (Kirby, 2006).

Previous research has also shed light on a number of potential moderators of the association between risk behavior and family disruption. For instance, the association depends heavily on parental involvement (Menning, 2006) and the quality of the parent-child relation (McArdle et al., 2002; Barfield-Cottledge, 2015), as these can buffer against the adverse effects of family disruption (Booth et al., 2010; Van Ryzin et al., 2012). In contrast, conflict between parents or parents and adolescents was positively associated with a variety of risks or risk behaviors, including substance use (Kristjansson et al., 2009; Vanassche et al., 2014), juvenile delinquency (Schroeder et al., 2010; Vanassche et al., 2014), aggression (Espelage et al., 2014), problem behavior (Fomby and Sennott, 2013), victimization (Jablonska and Lindberg, 2007), and sexual onset and frequency (Jordahl and Lohman, 2009; Haglund and Fehring, 2010; Madkour et al., 2010; Boislard and Poulin, 2011). More parental communication buffered adolescents against the negative influence of, for example, bullying (Ledwell and King, 2015). And father involvement proved to be a protective factor for risky sexual behavior (Jordahl and Lohman, 2009).

Previous research suffers from two shortcomings. One is the often exclusive focus on just one risk behavior although different risk behaviors seem to be connected through common pathways (Madkour et al., 2010; Espelage et al., 2014; Ttofi et al., 2014; Bozzini et al., 2021). Another shortcoming is the oftentimes exclusive focus on the first or main household of an adolescent. This focus ignores the cumulative impact of multiple family transitions and the complexity of resulting family structures, an aspect that has been highlighted by scholars in family demography, sociology, and biosocial family science on outcome differences between step- and biological parent-child ties (Schnettler and Steinbach, 2011; Thomson, 2014; Schnettler and Willführ, 2019). Although recently, new research designs have been applied to specifically oversample complex families and thus to take family complexity into account (e.g., Kalmijn et al., 2018), for most countries such data are still not yet available, thus limiting the statistical power to analyze rare types of (complex) families. Our investigation improves on both issues by analyzing the association of several risk behaviors at once (substance use, bullying, and early sexual onset) with a wider range of complex family constellations across households, including stepparents in the first and second household.

Much of the research on family disruption and risk behavior draws on the instability hypothesis. It states that stress mediates the effects of family transitions on developmental outcomes (Fomby and Cherlin, 2007; Hadfield et al., 2018). When parents separate, relocate, and possibly re-partner, children tend to grow up in family and household constellations with various degrees of complexity (Thomson, 2014). From the point of view of an adolescent, parental separation possibly implies residential relocation, the establishment of a second parental home, and/or less contact with one of the two biological parents. Re-partnering of one or both biological parents makes family structure even more complex as it implies the addition of stepparental ties, possibly in both parental homes. Why is this relevant? From the point of view of the instability hypothesis, the effects of multiple stressors can accumulate. Navigating complex family relationships adds another set of potential stressors (see, e.g., Schier, 2015; Forsberg et al., 2016): First, it is more demanding to organize life across two households as multi-locality, among other things, involves regular commuting between homes, keeping multiples schedules, and regularly transferring belongings between the two households. Second, communicating and negotiating the demands of parents living in either household may be emotionally taxing, a challenge that arguably becomes more difficult as additional relationships are involved, e.g., toward stepparents and stepsiblings in the main and secondary parental home. We thus propose an extended instability-complexity hypothesis: Multiple family transitions and the degree of complexity of cross-household family structures both provide independent and additive sources of stress that may cumulate and affect developmental outcomes of adolescents.

Although single family transitions have been studied extensively and with regard to multiple outcomes (e.g., Amato, 2001; Kristjansson et al., 2009; Amato and Anthony, 2014), what has been studied less is the overall degree of potential cumulation of risk behavior with increasing family complexity. In the present study, we provide a broad descriptive account on how risk behavior is distributed across adolescents living in diverse family structures that can be characterized by various degrees of prior (potentially stressful) transitions and (potentially stressful) degrees of complexity. If indeed, the extended instability-complexity hypothesis holds true, we expect that the prevalence of risk behavior across family types roughly follows a gradient with lower prevalence in family types with fewer preceding transitions and less complexity and higher prevalence in family types with a higher number of preceding transitions and/or more complexity.

With our data we are not able to test the causal effects of multiple transitions directly, that is, as they unfold over time. This would require detailed longitudinal data on the timing of these transitions and the timing of risk outcomes. But we are able to look at how the resulting states of such transitions (e.g., living in a family with separated vs. non-separated parents, with one or both biological parents re-partnered or not) along with indicators of family complexity are associated with various risk outcomes in adolescents. Unlike previous studies, our analysis is very detailed with regard to measuring family complexity as we are able to distinguish a much larger number of distinct family types than previous studies.

We also take into account the share of family types in a given country as a potential moderator of the association between family structure and risk-behavior in adolescents, which might provide leverage for policy-makers. The collective-declining-effect hypothesis predicts that the consequences of parental separation are less severe the more frequent relationship dissolution is in a given country (Albertini and Garriga, 2011). Drawing on this hypothesis, we assume that a higher proportion of non-traditional family types in a country could decrease the association between family complexity and adolescent risk behavior. Where post-separation family types are relatively frequent, this may reflect a regime with fewer normative sanctions against these families, with parental break-up being less selective, and family policies that provide support for the specific needs of them.

In sum, we want to improve on previous research addressing the association between family structure and adolescent risk behavior by looking at multiple risk behaviors rather than just one isolated risk behavior at a time, by considering information on both parental households in post-separation families, and by taking into account even rare family constellations. Drawing on previous research in the field of family and stress research, we derive two working hypothesis to be tested:

*Hypothesis 1: “The prevalence of risk behavior across family types roughly follows a gradient with lower prevalence in family types with fewer preceding transitions and less complexity, and higher prevalence in family types with a higher number of preceding transitions and/or more complexity.”*
*Hypothesis 2: “A higher proportion of non-traditional family types in a country decreases the association between family complexity and adolescent risk behavior as stated in Hypothesis 1.”*

## Materials and Methods

### Data

We use data from the nationally representative, cross-sectional “Health Behavior in School-aged Children” (HBSC) study, a WHO collaborative study with a focus on young people's well-being, health behavior, and their social context across countries and regions in Europe and North America (Currie et al., 2009; Richter, 2010). The survey was administered to adolescents aged 11, 13, and 15 years. These age groups “represent the onset of adolescence, a time when young people face the challenges of physical and emotional changes and important life and career decisions are beginning to be made” (Roberts et al., 2009, 47).

A strength of the HBSC study is that it uses a common research protocol and measurement at the same school age of adolescents, thus providing a degree of comparability not previously available for cross-national surveys on adolescent risk behavior and health (Currie et al., 2009, 132). Another strength is that all country surveys are representative samples using a cluster sampling design with school classes as sampling units wherever a suitable sampling frame was available, and schools and school classes within these schools wherever this was not the case (Roberts et al., 2009, 143). The study complied with high ethics and confidentiality requirements: In all cases, data were collected after ethical clearance was obtained by each participating country; active or passive consent from students and their legal guardians was required; and anonymity was ensured (Paakkari et al., 2020).

The HBSC network initially consisted of just three countries, but today more than fourty countries participate in this network (Currie and Alemán-Díaz, 2015). For the analysis, we pooled the cross-sectional data of the 2001, 2006, and 2010 waves of the HBSC study across 42 countries and regions.^1^ Although in each country, particular family types apply only to a small to medium percentage of all families, pooling over these countries and three waves allows us to study the association of growing up in a variety of complex family types and adolescent risk behavior. Altogether, the combined sample includes 581,838 adolescents.

### Measures

#### Risk Behavior

**Figure 1:**
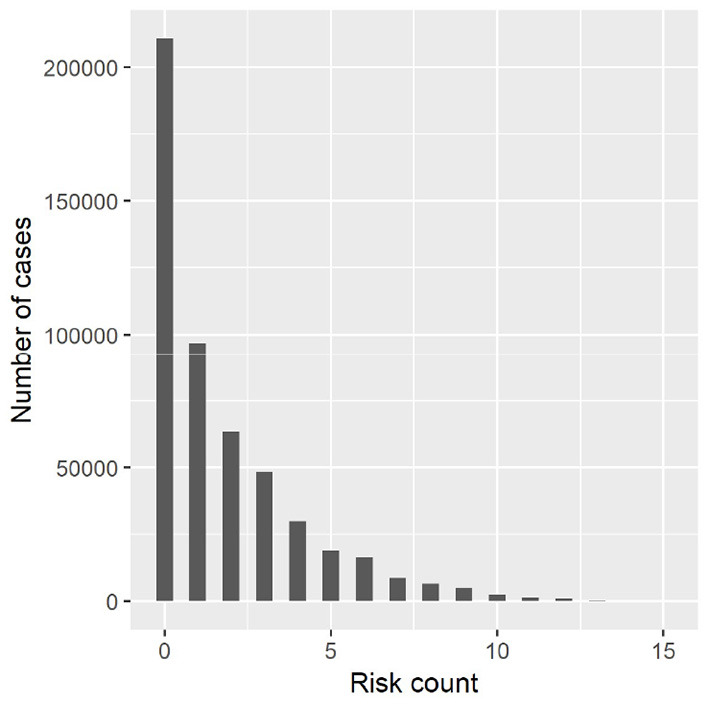
Histogram of risk count variable.

First, and reflecting the fact that single risk behaviors often do not occur in isolation (Bozzini et al., 2021), we created a count variable on the intensity of risk behavior. It is built from five indicators on the frequency of drinking, smoking, bullying perpetration, bullying victimization, and lifetime cannabis use, respectively, with various scales that we standardized to range from 0 (no engagement at all) to 3 (frequent engagement).^2^ The count variable sums up the scores from each of these five indicators and thus ranges from 0–15 (see Figure 1). For the overall risk count, we only took into account valid cases; that is, a missing value in one of the risk variables would not turn the overall count variable into a missing case. Second, we replicated the aggregate model for nine single risk indicators, taking into account the incidence of a risk by asking whether the respondent (1) bullied others, or (2) was bullied in the two month preceding the interview; (3) got into a physical fight or (4) contracted an injury in the past 12 months; ever tried (5) cannabis, (6) tobacco, or was (7) really drunk; (8) is currently smoking; and (9) ever had sex (“yes” = 1, “no” = 0). This allowed us to look at whether a broader set of risk behaviors was associated with family types.

#### Family Types

The household roster for the main and second home of the adolescent includes information on parents, stepparents, grandparents, and other persons living there or whether the respective home is a foster home. In a first step, we created family types based on the following inclusion rules: at least one biological parent needs to be present in the first household (home 1) and, if applicable, in the second household (home 2) of the respondent, and only (step-) parents are considered for the construction of family types. Although, in our models, we statistically control for the presence of grandparents and other individuals, we did not use this information to inform our choice of family types. This left 13 family types, ranging from the two-biological-parent family to complex stepfamilies with two households and a stepparent living in each of the two homes. Six of these 13 family types (except the two-biological-parent family) mirror the six other family types with the only difference that either the biological father or mother is the focal parent living in home 1. Thus, the 13 family types can be reduced to seven family types (F1-F7) plus one indicator accounting for this difference. When referring to the original 13 family types we reflect this by adding an additional letter “a” (biological mother focal) or “b” (biological father focal).

**Figure 2:**
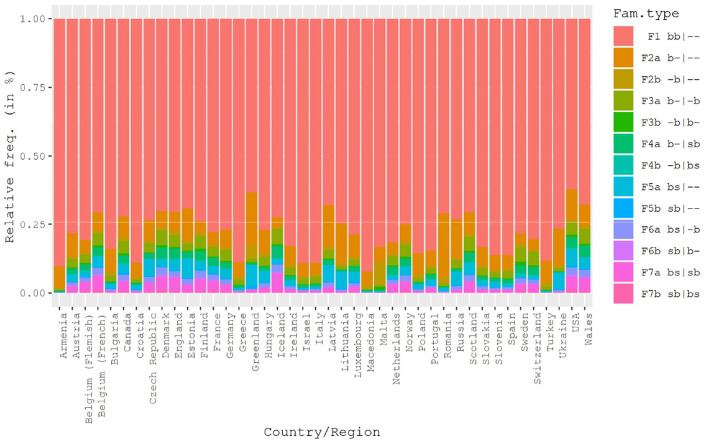
Relative frequencies of 13 family types within countries. The legend shows which type of parents live in the first and second household. The letter “b” stands for a “biological” parent, the letter “s” for “step” parent. Letters on the left-hand side of the vertical bar “|” indicate constellations in the first, and, if applicable, letters on the right-hand side in the second household of the adolescent. The first of two letters per household refers to the mother, the second one to the father. For instance, family type “F6b sb|b-” indicates that a stepmother and a biological father live in household 1 and a biological mother in household 2.

A higher order number for a family type roughly reflects a higher number of prior family transitions and/or family complexity. F1—the reference group in all models—comes without an additional letter as it indicates two-biological-parent families. F2a and F2b distinguish a single mother from a single father household (see Table 1; Figure 2). Thus, as compared to adolescents living in F1-type families, adolescents in this group have experienced parental separation and unavailability^3^ of one biological parent (two stressors). F3 refers to adolescents whose two biological parents live separately in two households without stepparents living in either household. Thus, the respective adolescent experienced parental separation and has to organize life across two parental households (two stressors). F4 stands for families in which the respective adolescent lives with one single biological parent in household 1 (HH1) and with the other biological parent and a stepparent in HH2. Thus, the respective adolescent in this constellation has experienced parental separation, re-partnering of one parent, and has to organize life across two households (three stressors). F5 stands for families in which the respective adolescent lives with only one biological parent and a stepparent in HH1 and there is no second parental home. Thus, the respective adolescent has experienced parental separation, parental re-partnering, and parental unavailability (three stressors). F6 stands for families in which the respective adolescent lives with a biological and stepparent in HH1 and a biological parent without a stepparent in HH2. Thus, these adolescents have experienced parental separation, re-partnering of one parent, and have to organize life across two households (three stressors). Finally, F7 stands for families in which two parental households are present and either biological parent lives with a new partner. Thus, adolescents in this family type have experienced parental separation, re-partnering of both parents, and have to organize life across two households (four stressors).

**Table 1:** Frequency distribution of the 13 family types.

	**Family type**		**Number of cases**	**%**
1	F1	bb|–	394,596	77.8
2	F2a	b-|–	38,345	7.6
3	F2b	-b|–	5,064	1.0
4	F3a	b-|-b	16,249	3.2
5	F3b	-b|b-	2,453	0.5
6	F4a	b-|sb	10,412	2.1
7	F4b	-b|bs	1,329	0.3
8	F5a	bs|–	14,477	2.9
9	F5b	sb|–	1,892	0.4
10	F6a	bs|-b	7,011	1.4
11	F6b	sb|b-	1,127	0.2
12	F7a	bs|sb	12,397	2.4
13	F7b	sb|bs	1,625	0.3
Total			506,977	100

We selected only adolescents living in one of these 13 types. From this initial selection we filtered out adolescents who, although associated with one of these family types, also responded to live in a foster home. Furthermore, we filtered out adolescents who said they had a second home but named no one living there and cases with missing values on any of the variables included in the multivariate models. This left us with a total of 506,977 adolescents. Table 1 shows the frequency distribution of family types for the remaining sample. The relative frequencies range between 0.2 and 77.8% or 1,127 to 394,596 cases. This illustrates that even very rare family types occur in relatively large absolute frequencies in the pooled data set. Figure 2 shows the relative frequencies of family types by country.

#### Control Variables

*Age* is operationalized as a categorical variable with the categories 11-, 13-, and 15-years of age. We further controlled for *survey year* (2002, 2006, and 2010), *gender* (“boy,” “girl”), whether a *grandfather, grandmother, or another person* lived in either home 1 or 2 (“yes,” “no”), the *share of specific family types* by country, whether adolescents spend 50% of the time in the second home (*joint physical custody*), and *socioeconomic status* of the family operationalized using the Family Affluence Scale (FAS1) (Currie et al., 2008). To account for differences in levels of risk behavior between countries, we used unconditional fixed effects models that account for the clustering of individuals within countries (Hilbe, 2009).

### Regression Models

We used count regression models to regress the count of risks dependent on family type and control variables. Different specifications were tested to determine the set of relevant control variables, family type variables and interaction effects, as well as to account for potential over-dispersion and excess zeros in the count models. Determined on the basis of the Bayesian Information Criterion (BIC), the best fit was obtained with a negative binomial count model that included the reduced set of family types in addition to an indicator for whether the father was the focal parent in home 1. The final model also included interaction effects between this indicator and family types, and gender of the adolescent as well as its interaction with all family type dummies.

## Results

**Figure 3:**
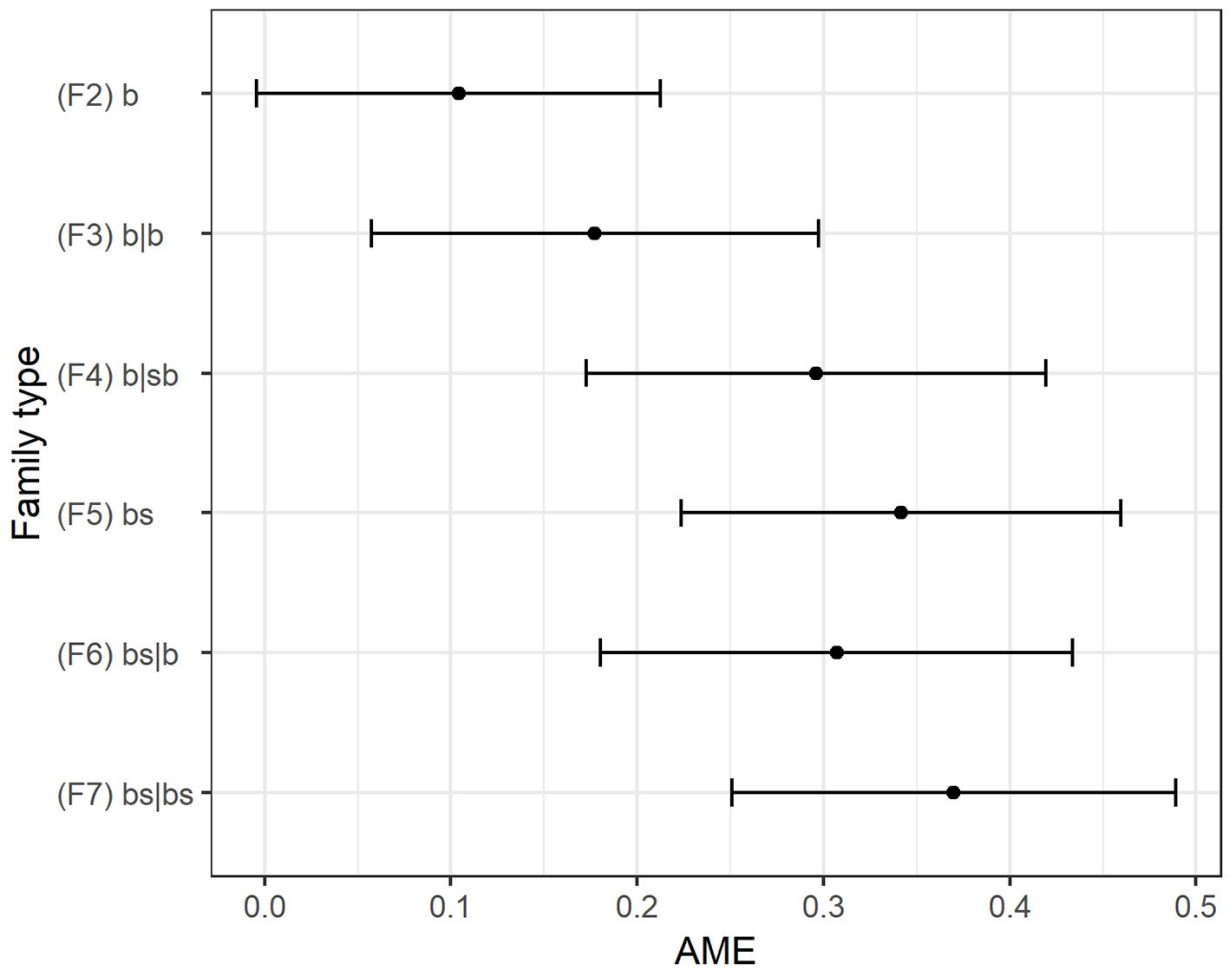
Average marginal effects of family type on risk count, reference group: two-biological-parent family (F1 bb).

For our final model,^4^ which is specified as a negative binomial (NB) count model to account for overdispersion, the association of family type and adolescent risk behavior is best illustrated by plotting the average marginal effects (AME) (Figure 3): These roughly increase with higher family complexity where complexity implies one or more of the following characteristics besides the fact that the biological parents don't live together in home 1: a second household exists and/or step- and biological parents co-reside in either one or two households. The coefficients for the final model show the same pattern as the margins plot: the expected log counts roughly increase with family complexity (see abbreviated regression Table 2, “Negative Binomial”). However, the differences between the family types are not as large as expected. On the contrary, they are rather small. As expected, joint physical custody and higher proportions of the respective family type in a country's population reduce the expected risk count.

**Table 2:** Abbreviated regression table: comparison of final, negative binomial count model (NB) with a negative binomial hurdle model (NBH) (some coefficients omitted from table).

	**NB**	**NBH (Count)**	**NBH (Zero)**
Intercept	−0.44^***^	−0.01	−0.88^***^
	(0.05)	(0.05)	(0.08)
F2	0.11^***^	0.13^***^	0.29^***^
	(0.03)	(0.03)	(0.05)
F3	0.17^***^	0.18^***^	0.39^***^
	(0.04)	(0.03)	(0.05)
F4	0.23^***^	0.22^***^	0.48^***^
	(0.04)	(0.03)	(0.06)
F5	0.27^***^	0.24^***^	0.60^***^
	(0.04)	(0.03)	(0.05)
F6	0.28^***^	0.25^***^	0.56^***^
	(0.04)	(0.03)	(0.06)
F7	0.29^***^	0.25^***^	0.60^***^
	(0.04)	(0.03)	(0.05)
Father focal	0.14^***^	0.11^***^	0.18^***^
	(0.02)	(0.01)	(0.03)
Gender (boy)	0.32^***^	0.18^***^	0.49^***^
	(0.00)	(0.00)	(0.01)
% fam.type	−0.19^***^	−0.05	0.00
	(0.05)	(0.04)	(0.08)
JPC (1=yes)	−0.08^***^	−0.05^**^	−0.16^***^
	(0.02)	(0.02)	(0.03)
F2:Father focal	−0.05^*^	−0.04	−0.09^*^
	(0.02)	(0.02)	(0.04)
F5:Father focal	−0.18^***^	−0.14^***^	−0.28^***^
	(0.03)	(0.03)	(0.06)
F2:male	−0.08^***^	−0.03^**^	−0.07^**^
	(0.01)	(0.01)	(0.02)
F3:male	−0.12^***^	−0.08^***^	−0.11^***^
	(0.02)	(0.02)	(0.03)
F4:male	−0.12^***^	−0.08^***^	−0.08
	(0.02)	(0.02)	(0.04)
F5:male	−0.14^***^	−0.06^**^	−0.15^***^
	(0.02)	(0.02)	(0.04)
F6:male	−0.19^***^	−0.12^***^	−0.19^***^
	(0.03)	(0.03)	(0.05)
F7:male	−0.16^***^	−0.10^***^	−0.17^***^
	(0.02)	(0.02)	(0.04)
AIC	1,747,935.60	1,728,537.02	
BIC	1,748,737.41		
Log Likelihood	−873,895.80	−864,125.51	
Deviance	537,628.08		
Num. obs.	506,977	506,977	

*** *p < 0.001*,

** *p < 0.01*,

* *p < 0.05; control variables included in the respective models but omitted from this table are: age, country, persons living in homes 1 and 2, survey year, family affluence score; the coefficients in the second and third column are from the same NBH model: “count” stands for the count component of the model, “zero” for the zero component of the model*.

To account for excess zero counts, we compared the final model with a negative binomial hurdle model (NBH) that, in addition to the count component like in the original model, includes a zero-component that reflects whether a respondent engaged in any risk behavior at all (thus has a non-zero value in the risk count) (Zeileis et al., 2008). Although part of one combined model, we report the respective coefficients in two distinct columns of Table 2. The NBH-model fits the data better than the NB-model,^5^ but the AME^6^ in Figure 3 are nevertheless based on the NB-model, as AME for hurdle models were not implemented in the software we were using. Given that the overall pattern reflected in the regression coefficients remained roughly the same, the AME for the NB-model still provide a good overall picture of the association between family type and the engagement in risk behavior. However, the coefficients of the hurdle model entail some additional information. They show that the same variables that are associated with risk severity in the count model are also associated with initiating risk behavior at all (the zero model). Joint physical custody reduces the expected log count of risks by a value of 0.05 in the count and the log odds by 0.16 in the zero component of the hurdle model. In the NBH model–and this is an important difference to the NB-model–the association between the country-specific proportions of family types is reduced in effect size and is not statistically significant.

**Figure 4:**
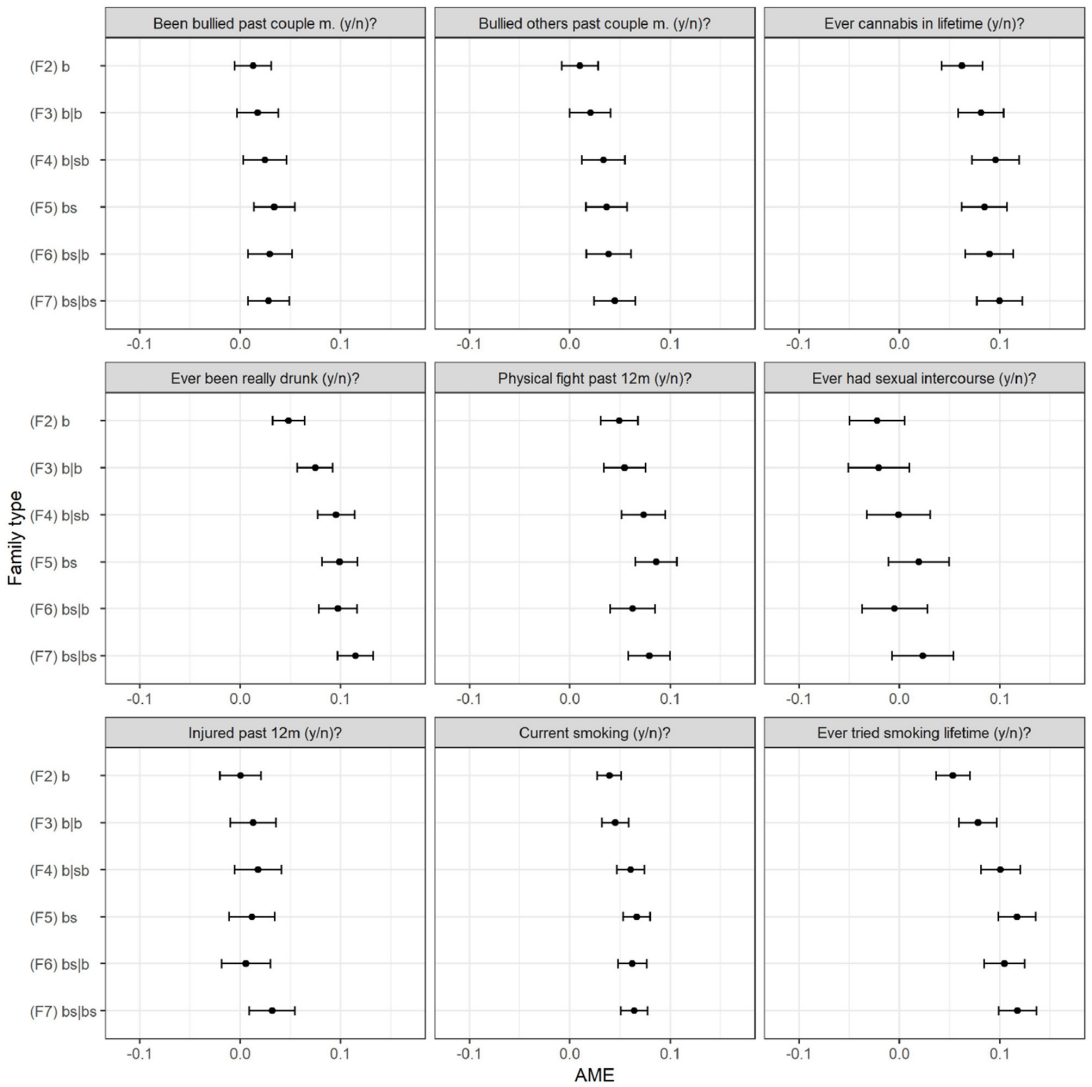
Average marginal effects of family type on risk behavior (9 separate indicators).

Finally, the results of separate logistic regression models in which we regress the risk of having engaged in any of nine different types of risks allow us to evaluate if the strength of the association between family type and adolescent risk behavior differs across types of risk behaviors. The association is illustrated in Figure 4 which shows the AME of family type across different indicators for risk behavior or exposure (see Supplementary Tables S2, S3 in the online supplement for the full regression tables of the respective models). We can see that on each of the nine indicators the basic patterns are the same as in the overall count model: the more complex the family type, the higher is the probability for risk behavior or exposure. However, the differences between family types, other than the difference with the two-biological parent family, are not as pronounced as expected. On the contrary, they are rather small (e.g., being drunk) or not existent (e.g., been bullied). Regarding our moderating factors, both joint physical custody and the proportion of family types show significantly negative coefficients for selected risk indicators.

## Discussion

Risk-taking behavior in adolescence has been found to be associated with a range of negative outcomes not only in adolescence but also at later ages (e.g., Hurrelmann and Richter, 2006). Previous research has identified family structure, itself associated with risk behavior, as a potential factor in the pathway affecting later-life outcomes (e.g., Amato, 2000; Brown and Rinelli, 2010; Härkönen, 2014). Our investigation extended previous research on adolescent risk behavior by comparing the association of a wide range of complex and even rare family types across households with two-biological families and by considering multiple types of risk behavior at once.

The results showed that in all family types other than the two-biological-parent family, adolescents show a higher probability of adopting risk behavior and a larger severity and breadth of risk behavior across different types of risks. As expected, this difference roughly follows a gradient with more complex family types being associated with a higher probability and intensity of risk behavior. This is consistent with the first of our two hypotheses stated in the introduction.

A key strength of our analysis is that by pooling data for multiple countries we have a considerably higher statistical power which allows us to uncover associations between risk behavior and even very rare family types that previous studies have neglected. However, our analysis is limited, as we cannot provide a causal analysis between measures of family complexity and risk behavior. This would require longitudinal data on the multiple set of transitions and other characteristics typical for complex families, and a considerable oversampling of rare family types. Unfortunately, data specifically covering complex post-separation family types are only available for selected countries (e.g., Kalmijn et al., 2018).

However, even though our findings are consistent with our first hypothesis, the expected differences between family types other than the two-biological-parent family are rather small. One reason may be that individuals differ in the way they experience cumulated stress. By focusing on risk behavior we look at just one set of potential outcomes, and thus the gradient in the association between outcome and family complexity might be rather weak. Our approach is in part an improvement compared to previous research that has mostly looked at single indicators of risk behavior when studying the potential effects of family transitions. By taking a broader view on multiple types of risk behavior simultaneously and thus acknowledging that risk behaviors often do not occur in isolation (Bozzini et al., 2021), we reveal a more pronounced complexity-risk-behavior gradient than in a series of models involving only isolated indicators of risk behavior. An even broader perspective, including other relevant outcomes that were previously studied in isolation, may reveal a stronger gradient. Relevant outcomes to be included in future research that were previously studied in isolation are, e.g., adolescent physical and mental health, suicidal ideation and attempts, and self-harming behavior (Härkönen, 2014; Bozzini et al., 2021).

Another reason for a weaker-than-expected association may be that we still oversee additional moderating effects in the association between risk behavior and family complexity. These could involve, for instance, indicators of relationship quality with parents, geographical proximity between the two parental households and thus the degree of difficulty organizing live across two household, and additional types of relationships. Most prominently, the latter could involve the presence and quality of complex sibling relationships, involving half- and step-siblings in one or both parental households. However, as relationship quality is likely to be associated with both risk behavior and other measures of family complexity, it will take more complex longitudinal research designs to disentangle these different associations.

In terms of potential moderating factors, our analysis showed that joint physical custody, although it does reduce the odds of adopting risk-taking behavior and its intensity, does not moderate the association between family type and the count of risks adolescents engage in. This may be due to the fact that our measure of joint physical custody is rather strict due to data limitations: we can only consider joint physical custody if adolescents spend about an equal amount of time in both homes. But our finding is consistent with other research using the same data that show that adolescents living in joint-physical-custody arrangements do not have significantly higher well-being as compared to adolescents living in other post-separation arrangements after controlling for child and other family characteristics (Steinbach et al., 2021).

In our second hypothesis we expected that the country-specific relative frequency of complex family types moderates the association between family complexity and risk behavior. This was justified with reference to the collective-declining-effect hypothesis. Yet, the empirical evidence for the moderating role of this variable is only tentative: Although we found a statistically significant risk-reducing effect in the negative binomial model, it almost disappeared in the better fitting negative binomial hurdle models. Then again, in selected logistic regression models on separate risk indicators, the main effect is significantly negative. That is, the odds for risk initiation decrease with an increasing proportion of the respective family type in a given country. A possible explanation for the instability of this effect may be due to its coarseness. Possibly, more fine-tuned and direct measures of family policies and norms across countries may be better suited to uncover potential moderating factors in the association of family type and adolescent risk behavior. Future research following up on this issue would benefit from an extended cross-national comparison that considers a wider set of contextual variables. The chosen variable on the proportion of family types is just one of many potentially relevant factors moderating the association between family complexity and risk behavior. Yet, a key limitation for comparative research, too, is that only few countries provide detailed (longitudinal) data on complex post-separation families using oversampling of family types that are rare in the general population.

We hope that our research is also of heuristic value for family researchers that want to better understand the mechanisms that lead to the described gradient in the probability and intensity of risk behavior with increasing family complexity. In the first part of this paper we suggested an extended instability-complexity hypothesis. But with our broad descriptive look at multiple risk behaviors and family types in a pooled, cross-sectional analysis, we are not able to directly test this hypothesis. This would require a more detailed look at specific parts of the suggested association and, as stated above, better, longitudinal data, including data on multiple transitions and measures of family complexity to cover even rare family types. Ideally, these data should become available for multiple countries in order to facility cross-country comparative research.

## Data Availability Statement

Publicly available datasets were analyzed in this study. This data can be found here: https://www.uib.no/en/hbscdata.

## Ethics Statement

Ethical review and approval was not required for the study on human participants in accordance with the local legislation and institutional requirements. Written informed consent for participation was not required for this study in accordance with the national legislation and the institutional requirements.

## Author Contributions

SS and AS conceived of the presented idea, prepared the paper outline, and wrote the introduction. SS conducted the data preparation and analysis. Both authors discussed the results and contributed to the final manuscript.

## Conflict of Interest

The authors declare that the research was conducted in the absence of any commercial or financial relationships that could be construed as a potential conflict of interest.

## Publisher's Note

All claims expressed in this article are solely those of the authors and do not necessarily represent those of their affiliated organizations, or those of the publisher, the editors and the reviewers. Any product that may be evaluated in this article, or claim that may be made by its manufacturer, is not guaranteed or endorsed by the publisher.

## Footnotes

^1^These include: Armenia (2010), Austria, Belgium (Flemish), Belgium (French), Bulgaria (2006), Canada, Croatia, Czech Republic, Denmark, England, Estonia, Finland, France, Germany, Greece, Greenland, Hungary, Iceland (2006, 2010), Ireland, Israel, Italy, Latvia, Lithuania, Luxembourg (2006, 2010), Macedonia, Malta (2002, 2006), Netherlands, Norway, Poland, Portugal, Romania (2006, 2010), Russia (2002, 2006), Scotland, Slovakia (2006, 2010), Slovenia, Spain, Sweden, Switzerland, Turkey (2006, 2010), Ukraine, USA, and Wales. If not otherwise noted in parentheses, all three waves (2002, 2006, and 2010) were available for the respective country.

^2^For smoking, the recoded scale ranges from 0 (“I do not smoke”) over 1 (“less than once a week”) and 2 (“once a week”) to 3 (“every day”). For drinking, the scale ranges from 0 (“never been drunk”) over 1 (“been drunk once”) and 2 (“been drunk two to ten times”) to 3 (“drunk more than ten times”). For bullying victimization and bullying perpetration, the respective recoded scales range from 0 (“haven't been bullied” or “haven't bullied in the past couple of months”) over 1 (“once or twice in the past couple of months”) and 2 (“(been) bullied two or three times per month”) to 3 (“once a week” to “several times a week”). For lifetime cannabis use, the recode scale ranges from 0 (“never taken cannabis”) over 1 (“once or twice”) and 2 (“three to nine times”) to 3 (“10 times or more”).

^3^We cannot infer from our data whether parental unavailability is due to parental death, hospitalization, departure, or any other reason.

^4^See Supplementary Table S1 in the online supplement for an overview of different model specification and the associated model fit to trace the process by which the final model was selected.

^5^The hurdle model was computed using the “hurdle()” function in the R package “pscl.” As this function doesn't provide BIC values for hurdle models, we based our decision on the AIC values instead.

^6^To compute AME we use the “margins” package in R.
