# Supplementary material for: Is Adolescent Risk Behavior Associated With Cross-Household Family Complexity? An Analysis of Post-separation Families in 42 Countries
Source: Front Sociol. 2022 Feb 16;7:802590. doi: 10.3389/fsoc.2022.802590 (PMC8888926; doi:10.3389/fsoc.2022.802590)
Supplement: Supplementary file 1 [file Table_1.DOCX]

Table S1: Model overview (included terms & BIC) for negative binomial count models, leading to final model (model 20)

| Model | F.types | Interactions | Cov. | Country FE | FT_Share | JPC | BIC |
| --- | --- | --- | --- | --- | --- | --- | --- |
| 1 | 13 | – | – | – | – | – | 1836010 |
| 2 | 7 | FT:FF | – | – | – | – | 1836010 |
| 3 | 7 | FT:FF(*) | – | – | – | – | 1835977 |
| 4 | 7 | FT:FF:Sex | – | – | – | – | 1830540 |
| 5 | 7 | FT:FF+FT:Sex(*) | – | – | – | – | 1830446 |
| 6 | 7 | – | yes | – | – | – | 1782119 |
| 7 | 7 | FT:FF | yes | – | – | – | 1782119 |
| 8 | 7 | FT:FF(*) | yes | – | – | – | 1782084 |
| 9 | 7 | FT:FF:Sex | yes | – | – | – | 1775652 |
| 10 | 7 | FT:FF+FT:Sex(*) | yes | – | – | – | 1775556 |
| 11 | 7 | – | yes | yes | – | – | 1755555 |
| 12 | 7 | FT:FF | yes | yes | – | – | 1755555 |
| 13 | 7 | FT:FF(*) | yes | yes | – | – | 1755520 |
| 14 | 7 | FT:FF:Sex | yes | yes | – | – | 1748858 |
| 15 | 7 | FT:FF+FT:Sex(*) | yes | yes | – | – | 1748764 |
| 16 | 7 | FT:FF+FT:Sex | yes | yes | %FT13 | – | 1748772 |
| 17 | 7 | FT:FF+FT:Sex | yes | yes | %FT7 | – | 1748759 |
| 18 | 7 | FT:FF+FT:Sex | yes | yes | %FT13:FF | – | 1748783 |
| 19 | 7 | FT:FF+FT:Sex | yes | yes | %FT7:FF | – | 1748771 |
| 20 | 7 | FT:FF+FT:Sex | yes | yes | %FT7 | JPC | 1748737 |
| 21 | 7 | FT:FF+FT:Sex | yes | yes | %FT7 | JPC:FF | 1748757 |
| 22 | 7 | FT:FF+FT:Sex | yes | yes | %FT7 | JPC:FT(*) | 1748782 |
| 23 | 7 | FT:FF+FT:Sex | yes | yes | %FT7 | JPC:FT:FF | 1748865 |
| 24 | 7 | FT:FF+FT:Sex | yes | yes | %FT7 | JPC:FT(*) | 1748757 |
| 25 | 7 | FT:FF+FT:Sex | yes | yes | %FT7 | JPC:FF:FT(*) | 1748791 |

Note: Table lists the BIC values for 25 models and helps to trace the process by which we decided on a final NB-model (Model 20). Dashed lines separate model blocks that differ by the addition of an additional term. The shaded lines indicate the model with the lowest BIC value per block. The columns further specify how the terms were added. “F.types” shows the number of family types in the model; “Interactions” which interaction terms between family type (FT), child gender (Sex), and whether the father is the focal biological parent in HH1 (FF) were included (* signals that only a statistically significant subset of the full combination of all factors was included); “Cov.” whether the variables age, year, grandmother/-father/other person resident in HH1/2, family affluence were added; “Country FE” the inclusion of country fixed effects; FT_Share which version of the variable indicating the country-specific percentage of family types was included in the model (here %FT13 is the share for 13 distinct family types, %FT7 the share of 7 distinct family types); and JPC how the variable indicating the joint physical custody arrangement was entered into the model.
