# Supplementary material for: Is Adolescent Risk Behavior Associated With Cross-Household Family Complexity? An Analysis of Post-separation Families in 42 Countries
Source: Front Sociol. 2022 Feb 16;7:802590. doi: 10.3389/fsoc.2022.802590 (PMC8888926; doi:10.3389/fsoc.2022.802590)
Supplement: Supplementary file 2 [file Table_2.DOCX]

TABLE S2: Logistic regression results risk initiation or exposure (9 different risk indicators) (part 1/2)

===========================================================================================================

(Model L1) (Model L2) (Model L3) (Model L4) (Model L5)

Ever Ever tried Smoking Ever tried Ever had

Drunk (y/n)? smoking (y/n)? now (y/n)? cannabis (y/n)? sex (y/n)?

-----------------------------------------------------------------------------------------------------------

Intercept -2.31 *** -3.27 *** -4.98 *** -6.92 *** -3.44 ***

(0.08) (0.09) (0.14) (0.28) (0.19)

Fam.type 2: b 0.37 *** 0.35 *** 0.42 *** 0.59 *** -0.08

(0.05) (0.05) (0.06) (0.09) (0.09)

Fam.type 3: b|b 0.60 *** 0.55 *** 0.58 *** 0.77 *** 0.12

(0.06) (0.06) (0.07) (0.11) (0.10)

Fam.type 4: b|sb 0.73 *** 0.70 *** 0.73 *** 0.99 *** 0.28 **

(0.06) (0.06) (0.07) (0.11) (0.11)

Fam.type 5: bs 0.73 *** 0.76 *** 0.71 *** 0.82 *** 0.29 **

(0.06) (0.06) (0.07) (0.10) (0.10)

Fam.type 6: bs|b 0.81 *** 0.77 *** 0.76 *** 0.98 *** 0.36 **

(0.07) (0.06) (0.08) (0.11) (0.11)

Fam.type 7: bs|bs 0.90 *** 0.85 *** 0.81 *** 0.99 *** 0.46 ***

(0.06) (0.06) (0.07) (0.10) (0.10)

Bio par. HH1: dad (y/n) 0.21 *** 0.27 *** 0.27 *** 0.22 *** 0.23 ***

(0.03) (0.03) (0.04) (0.05) (0.05)

Gender: male (Ref.: female) 0.39 *** 0.27 *** 0.12 *** 0.46 *** 0.48 ***

(0.01) (0.01) (0.01) (0.02) (0.01)

Age: 13y (Ref. 11y) 1.18 *** 1.37 *** 1.56 *** 1.59 *** 0.94 ***

(0.01) (0.01) (0.02) (0.08) (0.12)

Age: 15y (Ref. 11y) 2.50 *** 2.41 *** 2.71 *** 3.10 *** 2.42 ***

(0.01) (0.01) (0.02) (0.08) (0.12)

Fam. Affl. Scale 0.09 *** -0.00 -0.02 * 0.07 *** 0.05 ***

(0.01) (0.00) (0.01) (0.01) (0.01)

Survey year 2006 (v. 2002) -0.12 *** -0.38 *** -0.40 *** -0.24 *** -0.02

(0.01) (0.01) (0.01) (0.02) (0.02)

Survey year 2010 (v. 2002) -0.32 *** -0.58 *** -0.43 *** -0.38 *** -0.04 *

(0.01) (0.01) (0.01) (0.02) (0.02)

Grandmom in HH1? (yes=1) -0.02 -0.04 ** 0.00 -0.07 ** 0.00

(0.01) (0.01) (0.02) (0.03) (0.02)

Granddad in HH1? (yes=1) 0.03 -0.01 0.03 -0.06 0.06

(0.02) (0.02) (0.02) (0.03) (0.03)

Anyone else in HH1? (y/n) 0.14 *** 0.18 *** 0.18 *** 0.17 *** 0.16 ***

(0.02) (0.02) (0.03) (0.04) (0.04)

Grandmom in HH2? (y/n) 0.07 * 0.15 *** 0.12 *** 0.04 0.14 **

(0.03) (0.03) (0.04) (0.05) (0.05)

Granddad in HH2? (y/n) 0.04 -0.02 0.00 0.13 * -0.01

(0.04) (0.04) (0.04) (0.06) (0.06)

Anyone else in HH2? (y/n) 0.34 *** 0.33 *** 0.38 *** 0.46 *** 0.48 ***

(0.04) (0.04) (0.04) (0.06) (0.06)

Country-specific % fam. type 0.08 0.00 0.03 0.13 -0.83 ***

(0.08) (0.08) (0.10) (0.15) (0.14)

Joint physical custody (y/n) -0.03 -0.11 *** -0.07 0.02 -0.10

(0.03) (0.03) (0.04) (0.06) (0.06)

Fam.2: b x Bio par.HH1: dad -0.03 -0.13 ** -0.07 -0.07 -0.12

(0.05) (0.05) (0.05) (0.07) (0.07)

Fam.5: bs x Bio par.HH1: dad -0.29 *** -0.36 *** -0.33 *** -0.39 *** -0.29 **

(0.07) (0.06) (0.07) (0.10) (0.10)

Fam.2: b x Gender: male -0.13 *** -0.09 *** -0.04 -0.09 * -0.12 **

(0.02) (0.02) (0.03) (0.04) (0.04)

Fam.3: b|b x Gender: male -0.24 *** -0.19 *** -0.25 *** -0.13 * -0.48 ***

(0.04) (0.04) (0.04) (0.06) (0.06)

Fam.4: b|sb x Gender: male -0.23 *** -0.24 *** -0.24 *** -0.30 *** -0.54 ***

(0.05) (0.04) (0.05) (0.07) (0.07)

Fam.5: bs x Gender: male -0.18 *** -0.14 *** -0.04 -0.12 * -0.30 ***

(0.04) (0.04) (0.04) (0.06) (0.06)

Fam.6: sb|b x Gender: male -0.38 *** -0.33 *** -0.27 *** -0.38 *** -0.74 ***

(0.05) (0.05) (0.06) (0.08) (0.09)

Fam.7: sb|sb x Gender: male -0.33 *** -0.35 *** -0.32 *** -0.24 *** -0.59 ***

(0.04) (0.04) (0.05) (0.06) (0.06)

-------------------------------------------------------------------------------------------------------------

AIC 465423.87 517705.07 325634.91 161889.45 164927.02

BIC 466200.49 518483.59 326413.45 162609.38 165619.98

Log Likelihood -232641.93 -258782.53 -162747.45 -80874.73 -82394.51

Deviance 465283.87 517565.07 325494.91 161749.45 164789.02

Num. obs. 486286 499674 499806 216339 169891

===========================================================================================================

*** p < 0.001, ** p < 0.01, * p < 0.05
