# Supplementary material for: Is Adolescent Risk Behavior Associated With Cross-Household Family Complexity? An Analysis of Post-separation Families in 42 Countries
Source: Front Sociol. 2022 Feb 16;7:802590. doi: 10.3389/fsoc.2022.802590 (PMC8888926; doi:10.3389/fsoc.2022.802590)
Supplement: Supplementary file 3 [file Table_3.DOCX]

TABLE S3: Logistic regression results risk initiation or exposure (9 different risk indicators) (part 2/2)

====================================================================================================

(Model L6) (Model L7) (Model L8) (Model L9)

Been Bullied Been injured Been in fight

Bullied (y/n)? others (y/n)? past 12m (y/n)? past 12m (y/n)?

----------------------------------------------------------------------------------------------------

Intercept -1.71 *** -2.22 *** -0.77 *** -0.52 ***

(0.09) (0.09) (0.07) (0.08)

Fam.type 2: b 0.08 0.09 0.01 0.30 ***

(0.05) (0.05) (0.05) (0.05)

Fam.type 3: b|b 0.11 0.14 * 0.09 0.34 ***

(0.06) (0.06) (0.05) (0.06)

Fam.type 4: b|sb 0.12 * 0.18 ** 0.16 ** 0.51 ***

(0.06) (0.06) (0.05) (0.06)

Fam.type 5: bs 0.24 *** 0.28 *** 0.07 0.52 ***

(0.05) (0.05) (0.05) (0.05)

Fam.type 6: bs|b 0.19 ** 0.22 *** 0.09 0.40 ***

(0.06) (0.06) (0.06) (0.06)

Fam.type 7: bs|bs 0.19 *** 0.24 *** 0.17 *** 0.48 ***

(0.06) (0.06) (0.05) (0.06)

Bio par. HH1: dad (y/n) 0.08 ** 0.11 *** 0.01 0.17 ***

(0.03) (0.03) (0.03) (0.03)

Gender: male (Ref.: female) 0.21 *** 0.68 *** 0.47 *** 1.48 ***

(0.01) (0.01) (0.01) (0.01)

Age: 13y (Ref. 11y) -0.10 *** 0.35 *** 0.02 * -0.10 ***

(0.01) (0.01) (0.01) (0.01)

Age: 15y (Ref. 11y) -0.52 *** 0.27 *** -0.06 *** -0.41 ***

(0.01) (0.01) (0.01) (0.01)

Fam. Affl. Scale -0.08 *** 0.06 *** 0.18 *** 0.01

(0.00) (0.00) (0.00) (0.00)

Survey year 2006 (v. 2002) -0.09 *** -0.09 *** -0.10 *** 0.03 **

(0.01) (0.01) (0.01) (0.01)

Survey year 2010 (v. 2002) -0.18 *** -0.21 *** -0.10 *** -0.16 ***

(0.01) (0.01) (0.01) (0.01)

Grandmom in HH1? (yes=1) 0.04 *** -0.02 0.02 0.00

(0.01) (0.01) (0.01) (0.01)

Granddad in HH1? (yes=1) -0.01 -0.02 0.03 * 0.01

(0.02) (0.02) (0.01) (0.01)

Anyone else in HH1? (y/n) 0.10 *** 0.12 *** 0.09 *** 0.19 ***

(0.02) (0.02) (0.02) (0.02)

Grandmom in HH2? (y/n) 0.15 *** 0.17 *** 0.13 *** 0.20 ***

(0.03) (0.03) (0.03) (0.03)

Granddad in HH2? (y/n) 0.07 * 0.01 0.06 0.04

(0.03) (0.03) (0.03) (0.03)

Anyone else in HH2? (y/n) 0.20 *** 0.19 *** 0.25 *** 0.29 ***

(0.04) (0.04) (0.03) (0.04)

Country-specific % fam. type -0.09 -0.14 -0.15 * 0.05

(0.08) (0.08) (0.07) (0.08)

Joint physical custody (y/n) -0.13 *** -0.11 *** -0.05 -0.05

(0.03) (0.03) (0.03) (0.03)

Fam.2: b x Bio par.HH1: dad -0.01 -0.07 -0.02 -0.08

(0.04) (0.04) (0.04) (0.04)

Fam.5: bs x Bio par.HH1: dad -0.14 * -0.20 *** -0.12 * -0.16 **

(0.06) (0.06) (0.06) (0.06)

Fam.2: b x Gender: male -0.03 -0.06 ** -0.02 -0.10 ***

(0.02) (0.02) (0.02) (0.02)

Fam.3: b|b x Gender: male -0.04 -0.08 * -0.07 * -0.13 ***

(0.03) (0.03) (0.03) (0.03)

Fam.4: b|sb x Gender: male 0.02 -0.01 -0.17 *** -0.27 ***

(0.04) (0.04) (0.04) (0.04)

Fam.5: bs x Gender: male -0.12 *** -0.17 *** -0.04 -0.16 ***

(0.03) (0.03) (0.03) (0.03)

Fam.6: sb|b x Gender: male -0.08 -0.06 -0.13 ** -0.17 ***

(0.05) (0.05) (0.05) (0.05)

Fam.7: sb|sb x Gender: male -0.10 ** -0.03 -0.08 * -0.16 ***

(0.04) (0.04) (0.04) (0.04)

----------------------------------------------------------------------------------------------------

AIC 572966.04 573021.30 673619.14 585382.79

BIC 573754.21 573809.43 674409.59 586171.42

Log Likelihood -286412.02 -286439.65 -336738.57 -292620.40

Deviance 572824.04 572879.30 673477.14 585240.79

Num. obs. 489453 489148 505424 492581

====================================================================================================

*** p < 0.001, ** p < 0.01, * p < 0.05
